# Linc*HOTAIR* epigenetically silences *miR34a* by binding to PRC2 to promote the epithelial-to-mesenchymal transition in human gastric cancer

**DOI:** 10.1038/cddis.2015.150

**Published:** 2015-07-02

**Authors:** Y-w Liu, M Sun, R Xia, E-b Zhang, X-h Liu, Z-h Zhang, T-p Xu, W De, B-r Liu, Z-x Wang

**Affiliations:** 1 Department of Biochemistry and Molecular Biology, Nanjing Medical University, Nanjing, Jiangsu, PR China; 2 Department of Pathology, First Affiliated Hospital of Nanjing Medical University, Nanjing, Jiangsu, PR China; 3 Department of Oncology, Affiliated Drum Tower Hospital of Nanjing University, Nanjing, Jiangsu, PR China; 4 Department of Oncology, Second Affiliated Hospital of Nanjing Medical University, Nanjing, Jiangsu, PR China

## Abstract

lncRNAs play important roles in the epigenetic regulation of carcinogenesis and progression. Previous studies suggest that *HOTAIR* contributes to gastric cancer (GC) development, and the overexpression of *HOTAIR* predicts a poor prognosis. In this study, we found that *HOTAIR* was more highly expressed in diffuse-type GC than in intestinal type (*P*=0.048). In the diffuse type, there is significant relationship between *HOTAIR* expression and DFS (*P*<0.001). CDH1 was downregulated in diffuse-type GC tissues (*P*=0.0007) and showed a negative relationship with *HOTAIR* (*r*^2^=0.154, *P*=0.0354). In addition, *HOTAIR* knockdown significantly repressed migration, invasion and metastasis both in *vitro* and *vivo* and reversed the epithelial-to-mesenchymal transition in GC cells. We also showed that *HOTAIR* recruiting and binding to PRC2 epigenetically represses *miR34a*, which controls the targets C-Met (HGF/C-Met/Snail pathway) and Snail, thus contributing to GC cell-EMT process and accelerating tumor metastasis. Moreover, it is demonstrated that *HOTAIR* crosstalk with microRNAs during epigenetic regulation. Our results suggest that *HOTAIR* acts as an EMT regulator and may be a candidate prognostic biomarker and a target for new therapies in GC patients.

Gastric cancer is the fourth most frequent cancer and the second leading cause of cancer-related deaths worldwide.^1^ The poor prognosis of patients with gastric cancer is largely due to the high frequency of tumor recurrence or metastasis after surgical resection.^2^ Chemotherapy and molecularly targeted therapy are the main treatments for advanced gastric cancer. Therefore, a better understanding of the early events associated with gastric cancer metastasis is warranted to decrease mortality and improves patient's quality of life.

In the past decades, cell and tumor biologists have identified the key role of epithelial-mesenchymal transition (EMT) in cancer cell invasion and metastasis, a biological process where epithelial cells lose their polarity and undergo transition into a mesenchymal phenotype.^3^ Recent evidence revealed that EMT could enhance cancer cell invasion by promoting Rac-dependent mesenchymal migration, and also contributes to cancer cell proliferation and survival.^4, 5^ Generally, the important hallmarks of EMT include the loss of E-cadherin and increased expression of non-epithelial cadherins, such as vimentin and N-cadherin. The loss of E-cadherin expression is a fundamental event in EMT process and a crucial step in the progression of papillomas to invasive carcinomas.^6^ There are studies demonstrated that epigenetic changes, such as microRNAs (miRNAs), histone modifications and DNA methylation, are involved in cancer cell EMT.^7, 8, 9^ For example, *miR34a* inhibits the epithelial-to-mesenchymal transition and cancer cell migration.^10^ In the colorectal cancer *miR34a* enhances Snail expression and activates IL-6 R/STAT3 signaling to induce EMT.^11, 12^ Meanwhile, our previous study found that long non-coding RNA (lncRNA) *BANCR* contributes to non-small lung cancer cell invasion and metastasis via regulating EMT.^13^

It is estimated that 98% of the human genome transcripts are non-coding RNAs (ncRNAs), which form a highly complex regulatory network and have diverse biological functions in tumor genesis.^14^ lncRNAs are important new members of the ncRNA family that are greater than 200 nt without protein coding ability. Recently, researchers have linked the aberrant lncRNA expression with diverse human diseases, in particular cancers.^15, 16^ Therefore, identification of gastric cancer-associated lncRNAs and investigation of their molecular mechanisms in controlling EMT are important in understanding the molecular biology of gastric cancer metastasis and progression. The lncRNA HOX antisense intergenic RNA (*HOTAIR*) was first identified in 2007.^17^ Recently, lots of studies have shown that *HOTAIR* is overexpressed in colorectal cancer, pancreatic cancer, breast cancer and gastrointestinal stromal tumors and is positively correlated with a poor clinical outcome.^18, 19, 20, 21^ The activity of *HOTAIR* is partially due to its interaction with the polycomb repressive complex 2 (PRC2; EZH2, SUZ12 and EED), which enhances histone H3 lysine-27 trimethylation of the HOXD locus to decrease multiple gene expression from HOXD.^17^ Our previous study showed that *HOTAIR* expression is increased in gastric cancer tissues and is associated with malignant characteristics and poor prognosis. Furthermore, *HOTAIR* promotes gastric cancer cell proliferation *in vivo* and *in vitro* by competing ‘sponge' miR-331-3p.^22^ However, the molecular mechanisms of *HOTAIR* involved in gastric cancer cell metastasis remain largely unknown.

In this study, we found that *HOTAIR* is more highly expressed in diffuse-type gastric cancer than in intestinal-type gastric cancer and is negatively related to *E-cadherin*. High expression of *HOTAIR* in diffuse-type gastric cancer predicted poor DFS. Additional experiments revealed that *HOTAIR* knockdown significantly repressed migration, invasion and metastasis both *in vitro* and *in vivo* and reversed the gastric cancer cell EMT. In addition, *HOTAIR* also epigenetically downregulates *miR34a* by binding to PRC2 to activate its target genes C-Met (HGF/C-Met/Snail pathway) and Snail, thereby promoting EMT in advanced stages of gastric cancer. Our findings provide new insights into the mechanisms by which lncRNAs regulate the expression of miRNAs.

## Results

### HOTAIR and CDH1 expression levels in human gastric cancer tissue

**Figure 1:**
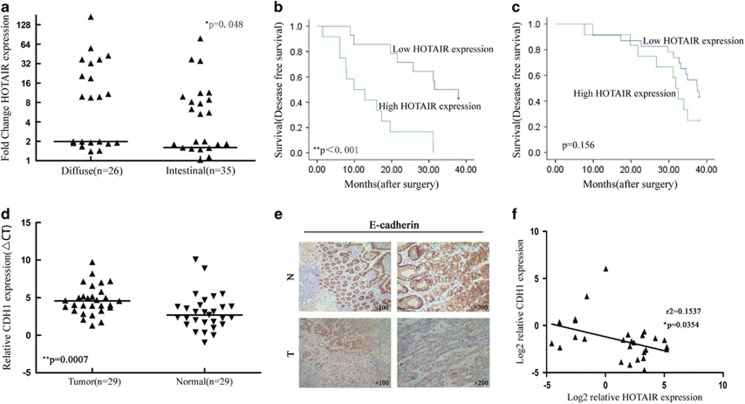
Relative *HOTAIR* expression in gastric cancer tissues and its clinical significance. (**a**) Relative expression of *HOTAIR* in the intestinal type of gastric cancer (*n*=35) compared to diffuse-type gastric cancer (*n*=26). *HOTAIR* expression was evaluated by qRT-PCR and normalized to GAPDH expression. Final results were presented as fold change in tumor tissues relative to normal tissues. Fold change is ≥2.0 for high expression and <2.0 for low expression. The data are presented as the fold change in the intestinal tissues relative to the diffuse tissues. (**b** and **c**) Kaplan–Meier disease-free survival curves according to the *HOTAIR* expression levels in different gastric cancer types. (**d**) Relative expression of CDH1 in diffuse-type gastric cancer compared to normal tissue (*n*=29). (**e**) Upper: E-cadherin immunostaining in normal tissue samples. Lower: E-cadherin immunostaining is negative in diffuse-type gastric cancer tissues. (**f**) Correlation analysis between the expression of *HOTAIR* and E-cadherin in 29 paired tumors. *P<0.05 and **P<0.01

The diffuse type has stronger metastasis behavior than the intestinal-type gastric cancer. We previously determined that *HOTAIR* expression was significantly upexpression in cancer tissues.^22^ In this study, the human gastric cancer tissues were histopathologically classified into intestinal (*n*=35) and diffuse types (*n*=26). *HOTAIR* expression was significantly higher in the diffuse-type gastric cancer (*P*=0.048) compared to the intestinal-type gastric cancer (Figure 1a). Examination of the correlation between *HOTAIR* expression and clinical pathological features showed that *HOTAIR* upregulation was correlated with lymph node metastasis and vasculature invasion (Table 1). For disease-free survival, patients with high *HOTAIR* expression had a significantly poorer prognosis than those with low *HOTAIR* expression for the diffuse-type gastric (*P*<0.001, log-rank test; Figure 1b); however, there was no significant correlation between *HOTAIR* expression and outcome for the intestinal-type gastric cancer (Figure 1c). *CDH1* is a vital metastasis marker in gastric cancer. We detected *CDH1 expression* by qPCR and immunohistochemistry. *CDH1* expression was downexpression in cancerous tissues (*P*<0.001) compared to the normal tissue, and was negative for 60% of the diffuse-type gastric cancer tissues (Figures 1d and e). In addition, *HOTAIR* was negatively correlated with *CDH1* (*r*^2^=0.154, *P*=0.0354; Figure 1f). These results indicate that *HOTAIR* overexpression play an important role in metastasis and may be useful for the development of novel prognostic or progression markers for advanced gastric cancer.

### Modulation of HOTAIR expression in gastric cancer cells

**Figure 2:**
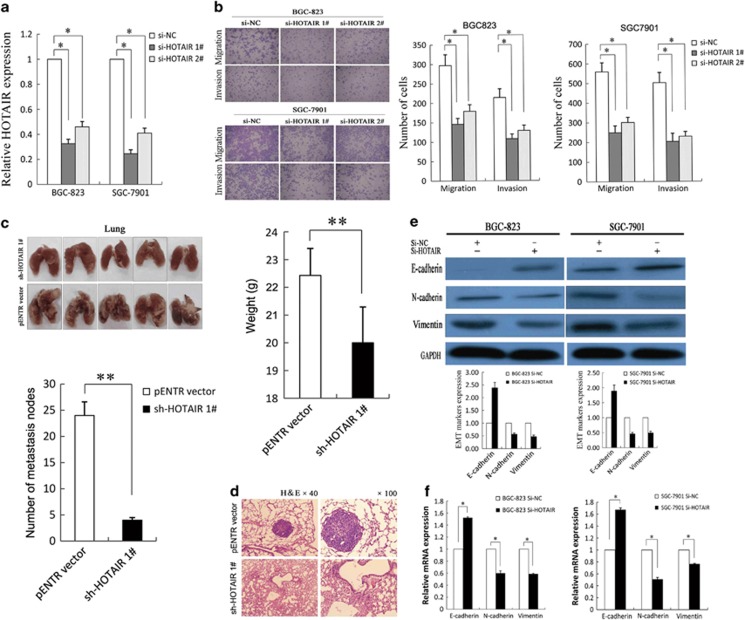
*HOTAIR*-promoted gastric cancer cell invasion, metastasis and EMT. (**a**) qRT-PCR was used to detect *HOTAIR* expression of BGC-823 and SGC-7901 cells with si-HOTAIRs. (**b**) Transwell assays were used to investigate the changes in migratory and invasive abilities of GC cells. (**c**) The lungs from mice in each experimental group with the numbers of tumor nodules on lung surfaces and weight were shown. (**d**) Visualization of the entire lung and HE-stained lung sections. (**e** and **f**) Western blotting and qRT-PCR were performed for analysis of E-cadherin, N-cadherin and vimentin in BGC-823 and SGC-7901 cells with si-HOTAIR. All experiments were performed in triplicate with three technical replicates. *P<0.05 and **P<0.01

To investigate the effect of *HOTAIR* on the invasion and metastasis of gastric cancer cells, we first examined the expression levels of *HOTAIR*, *miR34a* and EMT markers in various cancer cell lines by qRT-PCR. As shown in Supplementary Figure S1A, of the five gastric cancer cell lines (SGC-7901, BGC-823, MGC-803, AGS and MNK45), BGC-823 expressed higher levels of *HOTAIR* (4.11-fold) and lower levels of *miR34a* (0.11-fold) and *CDH1* (0.17-fold) than the normal gastric epithelium cell line (GES-1); however, SGC-7901 expressed relative lower *HOTAIR* and higher *miR34a* and *CDH1* expression. Therefore, we chose SGC-7901 and BGC-823 as the experimental cell lines. The results showed that *HOTAIR* expression was effectively knocked down in BGC-823 and SGC-7901 cells by si-HOTAIR1# and si-HOTAIR2# (Figure 2a), which were subsequently used in the further experiments. The efficiency of the sh-HOTAIR was shown in Supplementary Figure S2A.

### HOTAIR promotes gastric cancer cell invasion and metastasis *in vitro* and *in vivo*

To investigate the effect of *HOTAIR* on the gastric cancer cell migration and invasion, Transwell assays were performed and the results revealed that inhibition of *HOTAIR* decreased BGC-823 and SGC-7901 cell invasion and migration (Figure 2b). To investigate the metastasis-promoting function of *HOTAIR in vivo*, we used a tail vein assay. Transwell assays certificated that the sh-HOTAIR1# and sh-HOTAIR2# treatment both decreased the invasion and migration of BGC-823 cells (Supplementary Figure S2B). Western blot assays certificated that the sh-HOTAIR1# treatment increased the epithelial marker E-cadherin in BGC-823 cells (Supplementary Figure S2C). Furthermore, sh-HOTAIR stably transfected BGC-823 cells were injected mice tail vein. Seven weeks after injection, the mice were killed and the lung tissues were collected. As expected, the *sh-HOTAIR* group exhibited a lower frequency of lung metastases and displayed less weight loss compared to the pENTR vector group (Figure 2c). This difference was further confirmed following examination of the entire lungs by hematoxylin and eosin (HE) staining of lung sections (Figure 2d). Taken together, these results indicate that *HOTAIR* possesses metastasis-promoting activity and that its upregulation may facilitate the metastasis of gastric cancer cells.

### HOTAIR promotes metastasis of gastric cancer cells by inducing EMT

Next, we investigated how *HOTAIR* facilitates metastasis of gastric cancer cells. As EMT is a critical event that contributes to tumor metastasis, we evaluated the effect of *HOTAIR* on gastric cancer cell-EMT process. First, we observed that BGC-823 and SGC-7901 cells transfected with si-HOTAIR partly restored epithelial cell polarity showing cobblestone-like morphology (Supplementary Figure S1C). Then, we detected the mRNA and protein expression levels of molecular markers of EMT following *HOTAIR* knockdown. The expression of the epithelial marker E-cadherin (*CDH1*) was higher in *HOTAIR* knockdown cells compared to the control cells. By contrast, the mesenchymal markers, including N-cadherin and vimentin, were decreased in the *HOTAIR* knockdown BGC-823 and SGC-7901 cells (Figures 2e and f). Furthermore, we obtained the same results used stably transfected cells that the expression of E-cadherin was increased in sh-HOTAIR cells compared to the control cells; however, the N-cadherin and vimentin were decreased in sh-HOTAIR in BGC-823 cells (Supplementary Figure S2C). These results suggest that *HOTAIR* may promote gastric cancer cell metastasis by inducing EMT.

### HOTAIR downregulates the miR34a in gastric cancer

**Figure 3:**
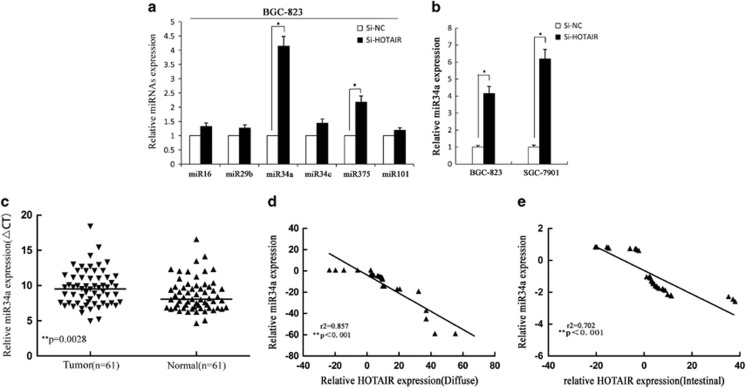
Correlation between the expression of *HOTAIR* and the *miR34a*. (**a** and **b**) *MiR34a* expression was detected in BGC-823 and SGC-7901 cells with si-HOTAIR by qRT-PCR. All experiments were performed in triplicate with three technical replicates. (**c**) The expression level of *miR34a* in 61 paired tumors and peritumoral gastric cancers was detected by qRT-PCR. (**d** and **e**) Correlation analysis between the expression of *HOTAIR* and *miR34a* was examined in diffuse- and intestinal-type gastric cancer tissues. *P<0.05 and **P<0.01

Recently, mounting evidences have shown that lncRNAs potentially regulate other classes of ncRNAs, including miRNAs. To investigate whether *HOTAIR* could regulate miRNA expression, we used Gene Set Enrichment Analysis (GSEA) software (Massachusetts Institute of Technology, Cambridge, MA, USA) to analyze the GES47638 data and predict the potential underlying miRNA of *HOTAIR* downstream. We found that various miRNAs may be *HOTAIR* downstream, and Supplementary Figure S1D shows target genes of six miRNAs, which were significantly enriched downstream of *HOTAIR*. Next, to investigate whether these miRNAs could be regulated by *HOTAIR* in gastric cancer cells, we subsequently detected miRNAs in si-*HOTAIR*-transfected BGC-823 or SGC-7901 cells and found that *miR34a* expression was upregulated by 4.2-fold compared with control cells (*P*<0.01, Figures 3a and b). Then we detected *miR34a* expression in gastric cancer tissues and found that *miR34a* expression was decreased in tumor tissue (Figure 3c) and negatively correlated with *HOTAIR* expression in diffuse and intestinal types (*r*^2^=0.857 and 0.702, *P*<0.001; Figures 3d and e). These data indicated that *HOTAIR* may downregulate *miR34a* expression and promote gastric cancer cell metastasis.

### HOTAIR silenced miR34a expression by recruiting PRC2

**Figure 4:**
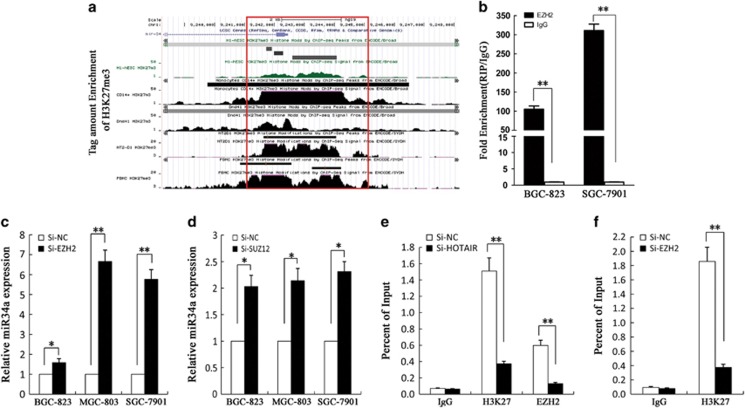
The association of *HOTAIR* with the PRC2 protein complex is critical for its regulation of *miR34a*. (**a**) Genome browser and analyzed H3K27me3 enrichment peaks in the *miR34a* promoter region. (**b**) RIP experiments were performed using the EZH2 antibodies for immunoprecipitation. Specific primers for *HOTAIR* were used to detect *HOTAIR*. (**c** and **d**) Expression of *miR34a* in BGC-823, SGC-7901 and MGC-803 cells transfected with si-EZH2, si-SUZ12 was detected by qRT-PCR. (**e** and **f**) ChIP analyses in SGC-7901 transfected with Si-HOTAIR and Si-EZH2 cells were performed on the *miR34a* promoter regions using anti-H3K27me3 and EZH2 antibodies. Enrichment was determined relative to the input controls. All experiments were performed in triplicate with three technical replicates. *P<0.05 and **P<0.01

It is reported that 20% of the lncRNAs can bind the polycomb group protein (PcG) complex to regulate downstream gene transcription. EZH2, a key subunit of PRC2, which also includes SUZ12 and EED is a histone methyltransferase and represses downstream gene transcription by trimethylating histone H3 lysine 27 (H3K27me3).^23, 24^ To determine whether *HOTAIR* regulates *miR34a* expression levels by binding with PRC2, we used ENCODE Histone Modification Tracks embedded in UCSC Genome Browser (UC Santa Cruz, CA, USA) and found H3K27me3 enrichment peaks in the *miR34a* promoter region (Figure 4a). Furthermore, we verified that *HOTAIR* was located both in the nucleus and cytoplasm of gastric cancer cells (Supplementary Figure S1B), and RNA immunoprecipitation (RIP) assays showed that *HOTAIR* could bind to PRC2 (Figure 4b). We knocked down EZH2 and SUZ12 by si-RNA in BGC-823, MGC-803 and SGC-7901 cells (Figures 4c and d) and demonstrated that *miR34a* was upregulated compared to the controls; however, *miR375* expression was not changed compared to control (Supplementary Figure S3A). The results of chromatin immunoprecipitation (ChIP) assays showed that EZH2 could directly bind to the promoter region of *miR34a* and mediate H3K27me3 modification, while knockdown of *HOTAIR* and *EZH2* led to reduced EZH2 and H3K27 binding ability (Figures 4e and f). In conclusion, these data indicate that *HOTAIR* recruit the PRC2 complex to silence *miR34a* via H3K27me3 modification.

### Upregulation of HOTAIR-enhanced gastric cancer cell metastasis via activating HGF/Met/Snail pathway

**Figure 5:**
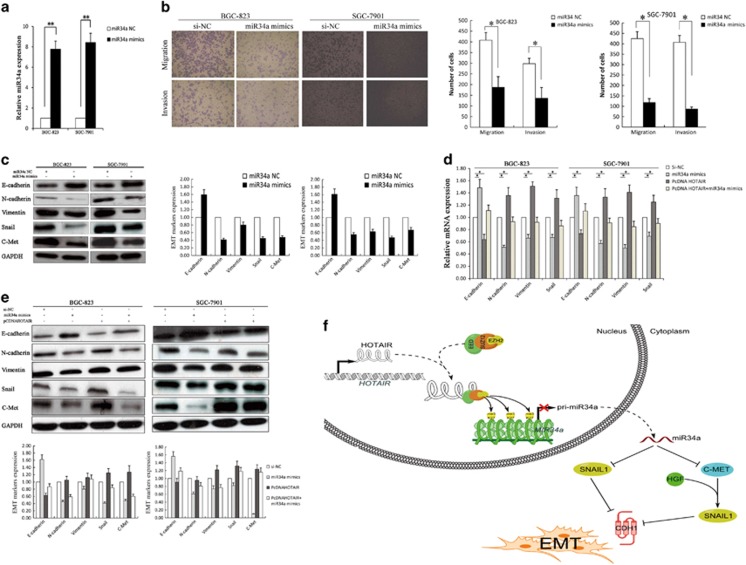
*HOTAIR* induces EMT by silencing *miR34a* in gastric cancer. (**a**) qRT-PCR was used to detect *miR34a* expression of BGC-823 and SGC-7901 cells with *miR34a* mimics. (**b**) Transwell assays were used to investigate the changes in the migratory and invasive abilities of gastric cancer cells with *miR34a* mimics. (**c**) Western blot assays were performed to detect the protein of C-Met, Snail and EMT markers in BGC-823 and SGC-7901 cells with *miR34a* mimics. GAPDH was used as a control. (**d** and **e**) Western blot assay and qRT-PCR were performed for analysis of E-cadherin, N-cadherin and vimentin; C-Met and Snail in BGC-823 and SGC-7901 cells. All experiments were performed in triplicate with three technical replicates. *P<0.05 and **P<0.01. (**f**) Binding of *HOTAIR* to PRC2 leads to epigenetically silencing *miR34a*, downexpression of *miR34a* facilitated C-Met transcription, which active HGF/C-Met/ to induce Snail gene transcription; in addition, *miR34a* directly regulates Snail translation, which repressed E-cadherin transcription to promote EMT in gastric cancer

Next, we investigated the role of *miR34a* in *HOTAIR*-induced EMT and metastasis promotion. *MiR34a* mimics were transfected into BGC-823 and SGC-7901 cells to upregulate *miR34a expression*, and the qPCR showed that *miR34a* expression was increased in *miR34a* mimics transfected gastric cancer cells (Figure 5a). Transwell assays revealed that the restoration of *miR34a* expression significantly inhibited the migration of BGC-823 and SGC-7901 cells (Figure 5b). HGF/Met/Snail pathway plays a critical role in EMT and metastasis, which was found to be regulated by *miR34a*.^25, 26, 27, 28^ Consistently, the restoration of *miR34a* in BGC-823 and SGC-7901 cells significantly reduced the protein levels of its targets C-Met and Snail, while upregulated E-cadherin and downregulated N-cadherin and vimentin (Figure 5c). Immunofluorescence analysis also revealed that enhanced *miR34a* expression decreased Snail expression not effected its subcellular localization in gastric cancer cells (Supplementary Figure S3C). By contrast, co-transfect of *miR34a* mimics and pcDNA-HOTAIR in BGC-823 and SGC-7901 cells partly reversed C-Met, Snail, E-cadherin, N-cadherin and vimentin expression (Figures 5d and e). These data suggest that

*HOTAIR* is mechanistically linked to increased gastric cancer cell metastasis via dependent of *miR34a*.

## Discussion

The incidence and mortality of gastric cancer have decreased significantly over the past 50 years worldwide; however, there remain large numbers of gastric cancer patients with poor prognosis for relapse and metastasis, particularly with diffuse-type gastric cancer in Asia.^29^ Recently, a study showed that nearly 76% of the GENCODE (The National Human Genome Research Institute, Bethesda, MD, USA) annotated lncRNAs was differentially expressed between gastric cancer and normal gastric tissue. This suggests that lncRNAs may play vital roles in carcinogenesis and may be useful for discovery of new biomarkers and therapeutic targets in gastric cancer.^30^ For example, *H19* is upregulated in gastric cancer and its overexpression is correlated with gastric cancer patient's poor prognosis, and enhances carcinogenesis and metastasis.^31^ In this study, our results revealed that high levels of *HOTAIR* expression were associated with venous invasion, diffuse-type gastric cancer and poor DFS. These findings suggest that *HOTAIR* has a vital role in aggressive and metastatic and may be a novel metastatic or prognostic marker for gastric cancer.

Recent studies have shown that EMT is an important molecular mechanism involved in cancer cell metastasis and progression. Hallmarks of EMT are the loss of E-cadherin expression and upregulation expression of N-cadherin and vimentin.^32, 33, 34, 35^ EMT is initiated by transcription factors or external signals, such as Snail, Twist, Zeb and HGF.^36, 37^ In addition to these signaling pathways triggered by membrane receptors, recent studies have highlighted the importance of ncRNAs in the regulation of the epithelial phenotype by controlling EMT inducers.^38^ *MiR200* is one of the major positive regulators in the maintenance of the epithelial phenotype via repression of ZEB1.^39^ Furthermore, the lncRNA *MALAT-1* promoted the EMT by regulating ZEB1, ZEB2 and Slug expression and activating Wnt signaling.^40^ In this study, we demonstrated that downregulation of *HOTAIR* expression contributes to the significant inhibition of cell migration, invasion and metastasis. Moreover, decreased levels of *HOTAIR* expression resulted in significant increased expression of E-cadherin but decreased expression of N-cadherin and vimentin in gastric cancer cells. These results demonstrate that *HOTAIR* mediates gastric cancer cell migration and metastasis, which also may be via affecting EMT.

Recently, mounting evidences have shown that lncRNAs potentially interact with other classes of ncRNAs, including miRNAs, and epigenetically regulate the expression of multiple genes, including those involved in chromatin modification, transcription and post-transcriptional processing. For example, *HOTAIR* functions as a ‘CeRNA' to regulate HER2 expression by sponging *miR-331-3p* to promote proliferation in gastric cancer.^22, 41, 42^ Approximately, 20% of the lncRNAs can recruit the PcG complexes to regulate gene expression.^43^ EZH2 could epigenetically silence the downstream gene transcription, and it is overexpressed in several types of cancer, including gastric cancer.^44, 45, 46^ In this study, we demonstrated that *HOTAIR* recruits and binds to PRC2 to epigenetically silence *miR34a* expression to promote gastric cancer cell-EMT process and metastasis.

In addition, *miR34a* expression was downregulated and negatively correlated with *HOTAIR* expression in gastric cancer tissues. The identified targets of *miR34a* include CDK4/6, cyclin E2 (CCNE2), cyclin D1 (CCND1), E2F3, c-Met and Snail,^47, 48, 49^ and HGF/C-Met /Snail pathway plays an important role in EMT and cancer cell metastasis.^27, 38, 50^ HGF binding to its receptor c-Met leads to autophosphorylation and then activate the Ras/Raf/MAPK pathway, which induces Snail gene transcription.^27^ Simultaneously, *miR34a* directly regulates Snail translation by binding to its 3′UTR and inhibiting protein coding.^11^ In this study, restoration of *miR34a* significantly inhibited the migration and invasion capacity of gastric cancer cells *in vitro*, and suppressed the EMT of gastric cancer cells by targeting Met and Snail. In addition, upregulation of *HOTAIR* could partially reversed *mi34a* overexpression-mediated C-Met, E-cadherin, N-cadherin, vimentin and snail expression.

LncRNAs have been proposed as potential targets for prognosis and therapeutic intervention. We describe here a novel mechanism by which *HOTAIR* reduced expression of *miR34a*: (i) *HOTAIR* recruited and binded to PRC2 epigenetically silencing *miR34a*; (ii) which in turn activate C-Met and as a consequence resulting in the activation of Snail transcription; and (iii) upregulated Snail expression promotes EMT in gastric cancer (Figure 5f). The prognostic significance of *HOTAIR* expression will help us to better predict the risk of recurrence. Finally, these data provide new insights into the RNA regulation network, indicating that lncRNAs not only target proteins but also affect miRNA expression through chromatin modification. Individual therapy targeting both *miR34a* and *HOTAIR* may lead to improved treatment.

## Materials and Methods

### Tissue samples

In this study, matched tumor tissues and adjacent non-tumor tissues were obtained from 61 gastric cancer patients at the Department of Surgical Oncology Jiangsu Province People's Hospital, Nanjing Medical University and Subei People's Hospital from March 2011 to December 2011. Two pathologists evaluated all specimens according to the World Health Organization (WHO) guidelines and the pTNM Union for International Cancer Control (UICC) pathological staging criteria. No local or systemic treatments were administered to these patients before surgery. The tissues were immediately frozen in liquid nitrogen and stored at −80 °C until use. Informed consent was obtained from all patients. The Human Research Ethics Committee of Jiangsu Province People's Hospital and Subei People's Hospital approved the study.

### Total RNA extraction quantitative real-time polymerase chain reaction

Total RNA was extracted from the cultured cells and frozen tissues using TRIzol reagent (Invitrogen, Karlsruhe, Germany) following the manufacturer's protocol. Quantitative real-time polymerase chain reaction (PCR) was performed to detect *HOTAIR* and *miR34a* using the PrimeScript RT reagent kit and SYBR Premix Ex Taq (TaKaRa, Dalian, China) according to the manufacturer's instructions. The results were normalized to the expression of glyceraldehyde-3-phosphate dehydrogenase (GAPDH) or U6. The specific primers used are presented in additional file 3: Supplementary Table S1. The qPCR and data collection were performed on ABI 7500 (Applied Biosystems, Carlsbad, CA, USA). The qPCR results were analyzed and expressed relative to the CT (threshold cycle) values and then converted to fold changes; 2.0-fold change was considered significant.^18^

### Plasmid generation

The *HOTAIR* sequence was synthesized and subcloned into the pCDNA3.1 (Invitrogen, Shanghai, China) vector. Ectopic expression of *HOTAIR* was achieved via pCDNA-HOTAIR transfection, with an empty pCDNA3.1 vector used as a control. We also synthesized shRNA sequence-targeted *HOTAIR*. Si-HOTAIR sequence removed five bases of the 3′ end were converted to sh-HOTAIR. After annealing of the complementary shRNA oligonucleotides, we cloned the annealed oligonucleotides into pENTR vector (*sh-HOTAIR*; additional file 3: Supplementary Table S1). The expression levels of *HOTAIR* were detected by qPCR.

### Immunohistochemistry

Paraffin-embedded, formalin-fixed human gastric tumor tissues were immunostained for the E-cadherin proteins. The signal was amplified and visualized using 3,30-diaminobenzidine chromogen followed by counterstaining with hematoxylin. Expression was considered positive when 10% or more of the cancer cells were stained. Anti-E-cadherin (1 : 50) was purchased from Cell Signaling Technology (CST, Danvers, MA, USA).

### Cell culture

The BGC-823 and MGC-803 lines were cultured in RPMI 1640 medium (Gibco, CA, USA) containing 10% fetal bovine serum and incubated at 37 °C, 5% CO_2_ and saturated humidity. The SGC-7901 cells were cultured in DMEM medium (Gibco, CA, USA) containing 10% fetal bovine serum and incubated at 37 °C, 5% CO_2_ and saturated humidity. Cell growth was observed under an inverted microscope. Cells in the logarithmic growth phase were harvested for the experiments.

### Cell transfection

Plasmid vectors (pCDNA3.1-HOTAIR and pCDNA3.1) for transfection were prepared using DNA Midiprep or Midiprep kits (Qiagen, Hilden, Germany) and transfected into BGC-823 or SGC-7901 cells. The si-HOTAIR, sh-HOTIR, si-EZH2, si-SUZ12, *miR34a* mimics or si-NC were transfected into BGC-823 and SGC-7901 cells (additional file 3: Supplementary Table S1). BGC-823 or SGC-7901 cells were grown on six-well plates to confluency and transfected using Lipofectamine 2000 (Invitrogen) according to the manufacturer's instructions. At 48 h post transfection, cells were harvested for qPCR or western blot analysis.

### Cell migration and invasion assays

For the migration assays, at 48 h post transfection, 5 × 10^4^ cells in serum-free media were placed into the upper chamber of an insert (8-*μ*m pore size; Millipore, Bedford, MA, USA). For the invasion assays, 1 × 10^5^ cells in the serum-free medium were placed into the upper chamber of an insert coated with Matrigel (Sigma-Aldrich, St. Louis, MO, USA). Medium containing 10% FBS was added to the lower chamber. After incubation for 24 h, the cells remaining on the upper membrane were removed with cotton wool. Cells that had migrated or invaded through the membrane were stained with methanol and 0.1% crystal violet, imaged and counted in five random fields per well using an IX71 inverted microscope (Olympus, Tokyo, Japan). Experiments were independently repeated three times.

### Western blotting analysis and antibodies

Cell lysates were prepared using RIPA protein extraction reagent (Beyotime, Beijing, China) supplemented with a protease inhibitor cocktail (Roche, Basel, Switzerland) and phenylmethylsulfonyl fluoride (Roche). Approximately, 50 *μ*g of protein extract was separated by 10% sodium dodecyl sulfate-polyacrylamide gel electrophoresis (SDS-PAGE) and then transferred onto a polyvinylidene fluoride (PVDF) membrane (Millipore). GAPDH was used as a control. Antibodies (1 : 1000) against E-cadherin and N-cadherin were purchased from BD (Franklin Lakes, NJ, USA). Antibodies (1 : 1000) against vimentin and C-MET were purchased from Cell Signaling Technology (CST). An antibody (1 : 1000) against Snail was purchased from Abcam (Cambridge, UK).

### Subcellular fractionation location

The separation of nuclear and cytosolic fractions was performed using the PARIS kit (Life Technologies, Grand Island, NY, USA) according to the manufacturer's instructions.

### Chromatin immunoprecipitation

We performed ChIP using the EZ ChIP chromatin immunoprecipitation kit for cell line samples (Millipore). Briefly, we sonicated the crosslinked chromatin DNA into 200- to 500-bp fragments. The chromatin was then immunoprecipitated using an anti-methyl-histone H3 antibody and EZH2 (1 : 5000). Normal mouse IgG was used as the negative control. The primer sequences are listed in Supplementary Table 1. The antibodies for the ChIP assays of EZH2 and H3K27 were obtained from Millipore. Quantification of the immunoprecipitated DNA was performed using qPCR with SYBR Green Mix (TaKaRa). The ChIP data were calculated as a percentage relative to the input DNA using the equation 2: input Ct−Target Ct × 0.1 × 100.

### RNA immunoprecipitation

We performed RIP experiments using the Magna RIP RNA-binding protein immunoprecipitation kit (Millipore) according to the manufacturer's instructions. The antibodies for the RIP assays of EZH2 were obtained from Abcam. The co-precipitated RNAs were detected by reverse-transcription PCR. The total RNAs were the input controls.

### Tail vein injections into athymic mice

We purchased athymic male mice (4 weeks old) from the Animal Center of the Chinese Academy of Science (Shanghai, China) and housed them in laminar flow cabinets under specific pathogen-free conditions. Sh-HOTAIR stably transfected BGC-823 cells were selected by using G418 (400 *μ*g/ml). BGC-823 cells transfected with HOTAIR1# and pENTR vector (EV) were harvested from six-well plates, washed with phosphate-buffered saline (PBS), and resuspended at 2 × 10^7^ cells per ml. The resuspended cells (0.1 ml) were injected into the tail veins of seven mice, which were killed 7 weeks after injection. The lungs were removed and photographed, and visible tumors on the lung surface were counted. This study was performed in strict accordance with the Guide for the Care and Use of Laboratory Animals of the National Institutes of Health. The Committee on the Ethics of Animal Experiments of Nanjing Medical University approved our protocol (Permit Number: 200933). All surgery was performed under sodium pentobarbital anesthesia, and all efforts were made to minimize suffering.

### Fluorescence immunohistochemistry

Cells were fixed in 4% paraformaldehyde following a standard protocol. A rabbit anti-Snail polyclonal antibody (1 : 50; Abcam) was used as a primary antibody, with TRITC-labeled anti-rabbit IgG (1 : 500; Sigma-Aldrich) used as a secondary antibody. Sections were mounted onto slides using Gel Mount aqueous mounting medium (G0918, Sigma-Aldrich) and examined with an Olympus BX51 microscope (Olympus Optical). Cells were fixed with 4% formaldehyde and examined by fluorescence microscopy to detect fluorescence intensity of the cells counted. Fluorescence intensity of the cells was measured using ImageJ (National Institutes of Health, Bethesda, MD, USA).

### Bioinformatics methods

GSEA software was downloaded from Broad Institute (http://www.broadinstitute.org/gsea/index.jsp). Gene profiling data downstream *HOTAIR* were obtained from Gene Expression Omnibus (GEO) site (http://www.ncbi.nlm.nih.gov/geo/query/acc.cgi?acc=GSE47638). Significantly enriched gene sets were identified, which produced a nominal *P*-value 0.05. UCSC Genome Browser (http://genome.ucsc.edu/cgi-bin/hgGateway) was used to analyze promoter regions.

### Statistical analysis

The SPSS 17.0 statistical analysis software (Armonk, NY, USA) was used for the statistical analysis of the experimental data. The significance of differences between groups was estimated by Student's *t*-test and the *χ*^2^ test. The levels of *HOTAIR* in the gastric cancer patients were compared using the Mann-Whitney *U* test. The levels of *HOTAIR*, *CDH1* and *miR34a* in the gastric cancer patients were assessed by the Spearman's correlation analysis. The disease-free survival probability was analyzed using Kaplan–Meier methods and evaluated using the log-rank test. A *P*-value <0.05 were considered significant.

## Figures and Tables

**Table 1:** Correlation of the expression of HOTAIR with clinicopathologic features in gastric cancer

**Characteristics**	***N* (%)**	**HOTAIR**^a^		***P*****-value**
		**High**	**Low**	
*Gender*				0.295
Male	38 (62)	17	21	
Female	23 (38)	7	16	
*Age*				0.605
≤65	31 (51)	11	20	
>65	30 (49)	13	17	
*Stage*				0.092
I–II	18 (30)	4	14	
III–IV	43 (70)	20	23	
*Lymph node metastasis*				0.05*
≤2	22 (36)	5	17	
>2	39 (64)	19	20	
*Vasculature invasion*				0.05*
Yes	28 (46)	18	10	
No	33 (54)	6	27	

*P<0.05 was considered significant (chi-square test between the two groups).

a Fold change (FC; tumor tissues relative to normal tissues) is ≥2.0 for high expression and <2.0 for low expression.

## Abbreviations

LncRNA: long non-coding RNA
HOTAIR: HOX antisense intergenic RNA
miR34a: microRNA34a
PRC2: polycomb repressive complex 2
EMT: epithelial-to-mesenchymal transition
PcG: polycomb group protein
BANCR: BRAF-activated non-coding RNA
ncRNA: non-coding RNA
EZH2: enhancer of zest homolog 2
H3K27me3: histone H3 lysine-27 trimethylation
HGF: hepatocyte growth factor
qRT-PCR: quantitative real-time polymerase chain reaction
DFS: disease-free survival
HE: hematoxylin and eosin
GSEA: gene set enrichment analysis
GEO: Gene Expression Omnibus
MALAT-1: metastasis-associated lung adenocarcinoma transcript 1
ZEB1: zinc-finger E-box-binding homeobox 1
ZEB2: zinc-finger E-box-binding homeobox 2
WHO: The World Health Organization
UICC: Union for International Cancer Control
GAPDH: glyceraldehyde-3-phosphate dehydrogenase
IHC: immunohistochemistry
SDS-PAGE: sodium dodecyl sulfate-polyacrylamide gel electrophoresis
PVDF: polyvinylidene fluoride
PMSF: phenylmethanesulfonyl fluoride
TBS: Tris-buffered saline
ChIP: chromatin immunoprecipitation
RIP: RNA immunoprecipitation
PBS: phosphate-buffered saline
DMEM: Dulbecco's modified Eagle's medium
CT: cycle threshold
BSA: bovine serum albumin
ECM: extracellular matrix
SiRNA: small interfering RNA
ShRNA: short hairpin RNA
GC: gastric cancer
IL6R: interleukin 6 receptor
STAT3: signal transducer and activator of transcription 3
TWIST: Transaction Workflow Innovation Standards Team
CDK: cyclin-dependent kinase
