# Supplementary figures and images for: LincHOTAIR epigenetically silences miR34a by binding to PRC2 to promote the epithelial-to-mesenchymal transition in human gastric cancer
Source: Cell Death Dis. 2015 Jul 2;6(7):e1802–. doi: 10.1038/cddis.2015.150 (PMC4650715; doi:10.1038/cddis.2015.150)

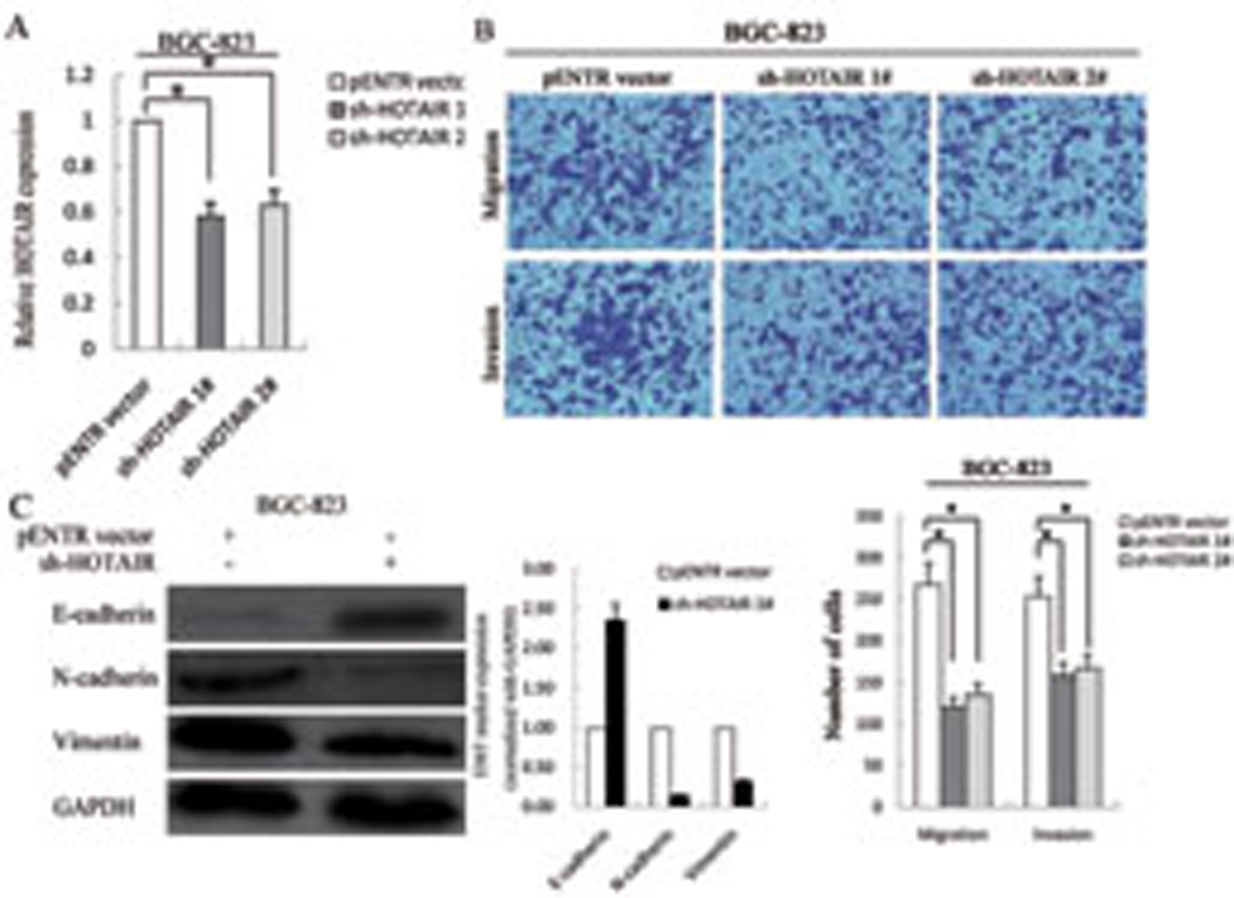

Supplement: Supplementary Figure S2 [file cddis2015150x2.tif]

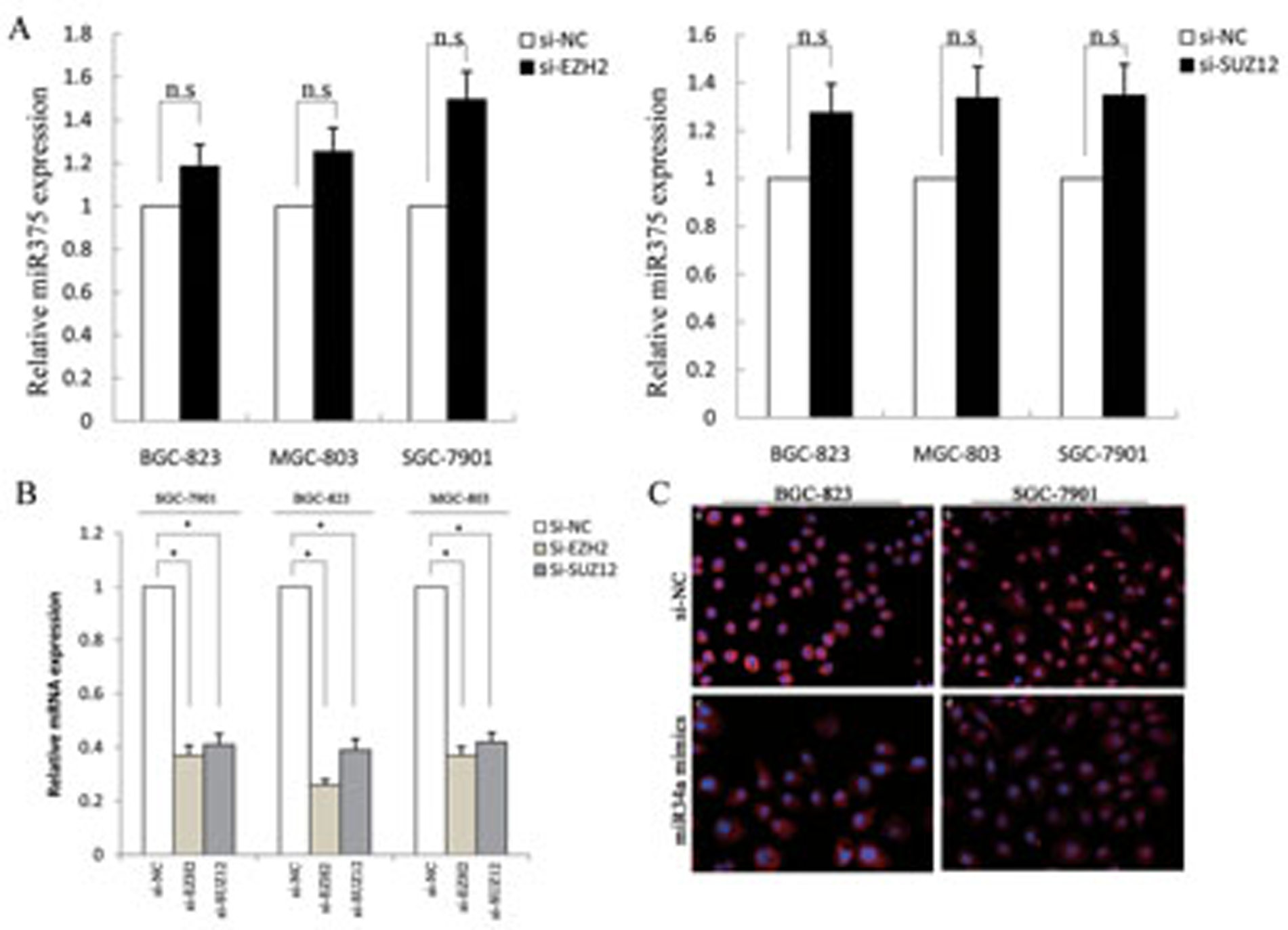

Supplement: Supplementary Figure S3 [file cddis2015150x3.tif]
