# Supplementary material for: LincHOTAIR epigenetically silences miR34a by binding to PRC2 to promote the epithelial-to-mesenchymal transition in human gastric cancer
Source: Cell Death Dis. 2015 Jul 2;6(7):e1802–. doi: 10.1038/cddis.2015.150 (PMC4650715; doi:10.1038/cddis.2015.150)
Supplement: Supplementary Figure Legends [file cddis2015150x4.doc]

**Supplement figure legends**

Fig S1 (A) The endogenous expression of *HOTAIR, miR34*a and EMT markers of GC cell lines (SGC-7901, MGC-803, BGC-823, AGS, MNK-45). (B) Fractionation of SGC-7901, BGC-823 and MGC-803 cells lysates demonstrates nuclear expression of *HOTAIR*. (C) The phenotypic characterization of BGC-823 and SGC-7901cells with si-HOTAIR on the EMT for 24h. (D) The GSEA results showed enrichment of several miRNAs that may be regulated by HOTAIR. The barcode plot indicates the position of the genes in each gene set, and red and blue represent positive or negative Pearson’s correlation with *HOTAIR* expression, respectively.

Fig S2 *HOTAIR* promoted gastric cancer cells invasion, metastasis and EMT. (A) qRT–PCR was used to detect *HOTAIR* expression of BGC-823 and SGC-7901 cells with sh-HOTAIR treatment. (B)Transwell assays were used to investigate the migratory and invasive abilities of BGC-823 cells with sh-HOTAIR treatment. (C) Western blot assays were performed to analyze E-cadherin, N-cadherin and Vimentin expression in BGC-823 cells with sh-*HOTAIR* treatment*.* *P < 0.05.

Fig S3 (A) Expression of EZH2 and SUZ12 in BGC-823, SGC-7901 and MGC-803 cells transfected with si-EZH2, si-SUZ12 was detected by qRT–PCR. (B) Analysis of *miR375* expression in BGC-823, MGC-803 and SGC-7901 cells with si-EZH2 or si-SUZ12 transfection. All experiments were performed in triplicate with three technical replicates. (C) Analysis of Snail in BGC-823and SGC-7901 cells with miR34a mimics transfection by immunofluorescence. *P < 0.05.
