# Supplementary material for: LincHOTAIR epigenetically silences miR34a by binding to PRC2 to promote the epithelial-to-mesenchymal transition in human gastric cancer
Source: Cell Death Dis. 2015 Jul 2;6(7):e1802–. doi: 10.1038/cddis.2015.150 (PMC4650715; doi:10.1038/cddis.2015.150)
Supplement: Supplementary Table S1 [file cddis2015150x5.doc]

Linc*HOTAIR* silences *miR34a* by binding to PRC2 to promote the epithelial-to- mesenchymal transition in human gastric cancer

Supplementary Table 1 The primer sequence of si-RNA,sh-RNA,pCDNA,mimics and promoter for qRT-PCR

| HOTAIR F | CAGTGGGGAACTCTGACTCG |
| --- | --- |
| HOTAIR R | GTGCCTGGTGCTCTCTTACC |
| E-cadherin F | TCCCATCAGCTGCCCAGAAA |
| E-cadherin R | TGACTCCTGTGTTCCTGTTA |
| N-cadherin F | CCCTGCTTCAGGCGTCTGTA |
| N-cadherin R | TGCTTGCATAATGCGATTTCACC |
| Vimentin F | TCTACGAGGAGGAGATGCGG |
| Vimentin R | GGTCAAGACGTGCCAGAGAC |
| Snail F | CCTGTCTGCGTGGGTTTT |
| Snail R | CTGAGGGTTCCTTGTGGG |
| GAPDH F | GGGAGCCAAAAGGGTCAT |
| GAPDH R | GAGTCCTTCCACGATACCAA |
| miR34aF | GGAGCAGCGAATCCACGATTAG |
| miR34aR | GAATGGTGGCAGTGTCTTAGC |
| miR34a RT | GCAGCGAATCCACGATTAGAACAACCAG |
| U6 F | CTCGCTTCGGCAGCACA |
| U6 R | AACGCTTCACGAATTTGCGT |
| si-EZH2 | GCUAAUGAGCUUCCAUACAATT |
| si-SUZ12 | UCGCCUGGCAUUGACCCAUTT |
| si-HOTAIR(1) | 5’-AAAUCCAGAACCCUCUGACAUUUGC-3’ |
| si-HOTAIR(2) | 5’-UUAAGUCUAGGAAUCAGCACGAAGC-3’ |
| si-HOTAIR(3) | 5’-CAUAUUAUAGAGUUGCUCUGUGCUG-3’ |
| pCDNA3.1-HOTAIR forward: | 5’-CATGGATCCACATTCTGCCCTGATTTCCGGAACC-3’ |
| pCDNA3.1-HOTAIR reverse: | 5’-ACTCTCGAGCCACCACACACACACAACCTACAC-3’ |
| sh-HOTAIR 1# sense | 5'-CACCG^*^AAATCCAGAACCCTCTGACACGAATGTCAGAGGGTTCTGGA-3' |
| sh-HOTAIR 1# anti-sense | 5'-AAAATCCAGAACCCTCTGACATTCGTGTCAGAGGGTTCTGGATTTC^*^-3' |
| sh-HOTAIR 2# sense | 5'-CACCG^*^TTAAGTCTAGGAATCAGCACCGAAGTGCTGATTCCTAGACTTAA-3' |
| sh-HOTAIR 2# anti-sense | 5'-AAAATTAAGTCTAGGAATCAGCACTTCGGTGCTGATTCCTAGACTTAAC^*^-3' |
|  | **ChIP-qRT-PCR primers** |
| miR34a2 F | CAGCATGTCTGCGTGAAAGG |
| miR34a2 R | GATCCTCTCCCTAAACGGTGC |
